# Scaling the phase-planes of social dilemma strengths shows game-class changes in the five rules governing the evolution of cooperation

**DOI:** 10.1098/rsos.181085

**Published:** 2018-10-17

**Authors:** Hiromu Ito, Jun Tanimoto

**Affiliations:** 1 Department of General Systems Studies, Graduate School of Arts and Sciences, University of Tokyo, Tokyo 153-8902, Japan; 2 Department of International Health, Institute of Tropical Medicine, Nagasaki University, Nagasaki 852-8523, Japan; 3 Department of Environmental Sciences, Zoology, University of Basel, 4051 Basel, Switzerland; 4 Department of Energy and Environmental Engineering, Interdisciplinary Graduate School of Engineering Sciences, Kyushu University, Fukuoka 816-8580, Japan; 5 Department of Advanced Environmental Science and Engineering, Faculty of Engineering Sciences, Kyushu University, Fukuoka 816-8580, Japan

**Keywords:** evolutionary games, scaling parameters, reciprocity, altruism, social viscosity

## Abstract

Game theory has been extensively applied to help explain how cooperative behaviour is promoted in human and animal societies. How do humans and animals establish reciprocity when confronting a social dilemma? In 2006, Nowak theoretically proved that numerous mechanisms and models for evolving cooperative behaviour reported over the last few decades can be distilled into five reciprocity mechanisms (rules). Additionally, universal scaling parameters were proposed to measure two different types of dilemmas, namely, the gamble-intending dilemma (GID) and risk-averting dilemma (RAD). In this work, by drawing a RAD–GID phase-plane diagram for pair-wise games, we prove that these five rules are indeed quite different for the resolution (relaxation) of the two dilemmas. These diagrams also demonstrate whether and when game-class change (resolution of a dilemma) occurs, thus implying how defectors can be eliminated.

## 1. Introduction

Game theory has been formulated as a tool for the optimization of individual behaviours and has been applied in biology to investigate the reasons why cooperative behaviour evolved in animal and human societies [1–6]. In game theory, the primary focus of the 2 × 2 (pair-wise) dilemma game is the investigation of the types of reciprocity mechanisms that enable players to overcome conflicts of interest and promote cooperative behaviours [7,8]. Social dilemma can be observed in various forms in the real world. Even if he/she does not know the word ‘prisoner's dilemma', he/she is exposed to various social dilemmas that are familiar to them. For example, people driving a car are exposed to the social dilemma when changing or merging their driving lanes [9,10]. Many people wonder whether to receive an expensive vaccination or not, because if people around them are vaccinated, the infection risk can be lowered without paying the vaccination fee (cost) [6,11,12]. Thus, understanding these social dilemma structures is important for us to construct a better cooperative society. In a seminal paper, Nowak presented a theory of reciprocity mechanisms for the evolution of cooperation in which all reciprocity can be categorized into the following five rules (protocols): direct reciprocity, indirect reciprocity, kin selection, group selection and network reciprocity [13]. These fundamental mechanisms are collectively known as ‘social viscosity'. The dilemma resolutions used in many (if not most) previous simulation studies can be categorized into one of these five protocols (rules) of reciprocity, and the five rules can be converted mathematically into a 2 × 2 payoff matrix [8]. These five reciprocity mechanisms and classical dilemma games (i.e. prisoner's dilemma game, chicken game (also hawk–dove game and snowdrift game) and stag–hunt game) have been extensively studied to understand the mechanisms of promotion and the evolution of cooperative behaviour [14–17]. All five reciprocity mechanisms are known to promote cooperative behaviour by resolution (relaxation) of social dilemmas. If we can visualize the relaxation of social dilemma which is mentioned above, it will help us to intuitively understand the evolution and promotion mechanisms of cooperative behaviour.

In this work, we denote the payoff matrix of pair-wise games with two strategies: cooperation (C) and defection (D). Player rewards are determined by the payoff matrix and the strategies that they choose (i.e. (C) or (D)) as follows:

1.1 $$A\equiv [a_{ij}]=\begin{array}{cc} & \begin{array}{cc} C & D \end{array}\\ \begin{array}{c} C\\ D \end{array} & \left(\begin{array}{cc} R & S\\ T & P \end{array}\right) \end{array},$$

where we consider an unlimited well-mixed population. In a pair-wise game, the strength of a dilemma that disturbs the promotion of cooperation is expressed as two types of universal scaling parameters: (i) the strength of the gamble-intending dilemma (GID) *D_g_*′ and (ii) the strength of the risk-averting dilemma (RAD) *D_r_*′ [12,18–20]. GID situation means that no solution is optimal in terms of the upper-side payoff, inevitably leading to a particular dilemma in which equal players are inclined to exploit each other. Therefore, the players' mutual strategy pairs (C, C) cannot be an equilibrium in GID situations. On the other hand, RAD situation means that there is no worst payoff on the lower side, inevitably leading to another particular dilemma, wherein equal players try not to be exploited by each other. Therefore, the players' mutual strategy pairs (D, D) must be an equilibrium [18]. In practice, *D_g_*′ appears because players attempt to exploit their opponents, while *D_r_*′ appears because players attempt to avoid exploitation by their opponents. These scaling parameters of dilemma strength depend on the relative magnitudes of the payoff matrix elements *P*, *R*, *S* and *T* (see equation (1.1)). Note that *x_i_*(*t*) is the frequency of strategy *i* at time *t*. The expected payoff for strategy *i* is given by $f_{i}=\sum_{\;j=1}^{2}x_{j}a_{ij}$. Therefore, the average payoff is given by $\varphi =\sum_{i=1}^{2}x_{i}f_{i}$. The replicator dynamics can be written as follows:

1.2 $${\dot{x}}_{i}=x_{i}(\;f_{i}-\varphi ).$$

For simplicity, if *x* (0 ≤ *x* ≤ 1) represents the fraction of cooperation strategy (C), the equilibrium of equation (1.2) is expressed in one or two of the following states:

1.3 $$x^{*}=0,\;1,\;\frac{P-S}{R-S-T+P}.$$

However, this condition does not hold in a finite well-mixed population or a population with any of the reciprocity mechanisms. If the game is depicted using classical GID (*D_g_* = *T* − *R*) and classical RAD (*D_r_* = *P* − *S*) dilemmas [18], the payoff matrix takes the following form:

1.4 $$A\equiv [a_{ij}]=\left(\begin{array}{cc} R & P-D_{r}\\ R+D_{g} & P \end{array}\right).$$

We also note that the third equilibrium of equation (1.3), which is the so-called internal equilibrium, can be represented as follows:

1.5 $$x^{*}=\frac{D_{r}}{D_{r}-D_{g}}.$$

These *D_g_* and *D_r_* are useful in quantifying the dilemma strength in games with an infinite well-mixed population [18]. However, in 2009, Tanimoto showed that these classical GID and RAD alone may be insufficient for indicating the dilemma strength when a specific reciprocity mechanism is introduced into a pair-wise game [19]. For this reason, Wang *et al*. introduce a new set of scaling parameters considering a finite population with any of the reciprocity mechanisms by defining a new set of GID and RAD as *D_g_*′ and *D_r_*′, respectively [20]:

1.6 $$D_{g}^{\prime }=\frac{T-R}{R-P}=\frac{D_{g}}{R-P}$$

and

1.7 $$D_{r}^{\prime }=\frac{P-S}{R-P}=\frac{D_{r}}{R-P}.$$

We refer to this definition as the new universal scaling for dilemma strength. Correspondingly, the payoff matrix is rescaled as follows:

1.8 $$A^{\prime }\equiv [{a^{\prime }}_{ij}]=\left(\begin{array}{cc} R & P-(R-P)D_{r}\\ R+(R-P)D_{g} & P \end{array}\right).$$

Note that the following equations hold by definition:

1.9 $$T=R+(R-P)D_{g}^{\prime }$$

and

1.10 $$S=P-(R-P)D_{r}^{\prime }.$$

**Figure 1.**
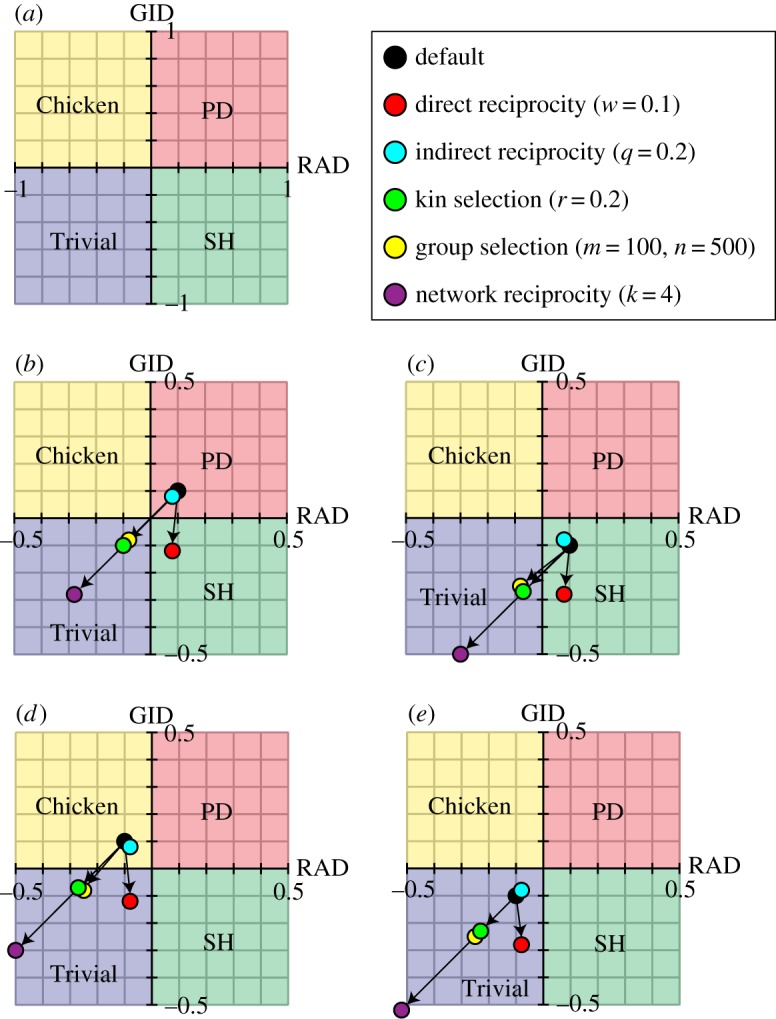
Phase planes of a pair-wise game with coordinate movements with the introduction of the five reciprocity rules. The shaded colours indicate the regions of Trivial (blue), Prisoner's dilemma (PD) (red), Chicken (yellow) and Stag-hunt (SH) (green) games. (*a*) Default RAD–GID phase plane. (*b*–*e*) Phase plane with coordinate movements according to the five rules. The default point (black circle) moves to each point (see above keys) with the introduction of the five rules. Initial default coordinates (*D_r_*′, *D_g_*′) are (*b*) (0.1, 0.1) in PD, (*c*) (0.1, −0.1) in SH, (*d*) (−0.1, 0.1) in Chicken and (*e*) (−0.1, −0.1) in Trivial.

**Figure 2.**
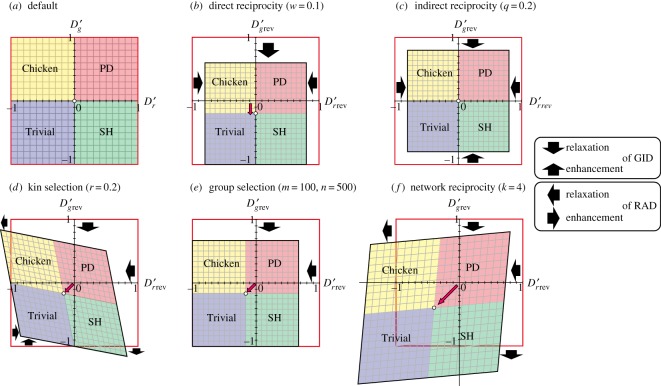
Transformation of the default phase plane (–1 ≤ *D_g_*′, *D_r_*′ ≤ +1) with the introduction of the five rules in all 2 × 2 games. The shaded colours indicate the regions of Trivial (blue), Prisoner's dilemma (PD) (red), Chicken (yellow) and Stag-hunt (SH) (green) games. (*a*) Default phase plane. (*b*–*f*) Transformed phase plane with the introduction of (*b*) direct reciprocity, (*c*) indirect reciprocity, (*d*) kin selection, (*e*) group selection and (*f*) network reciprocity. The origin in the default moves to the direction of the points indicated by pink arrows. The red frames indicate the default phase planes. The thick black arrows indicate the relaxation and enhancement of GID and RAD.

Depending on these two dilemma strengths, the game can be divided into four classes: Prisoner's dilemma (PD), Chicken (also known as the snowdrift or hawk–dove game), Stag-hunt (SH) and the Trivial game with no dilemma (electronic supplementary material, table S1) [12,20]. If both *D_g_*′ and *D_r_*′ are positive, the game is PD, whereby (D) dominates (C). If *D_g_*′ is positive and *D_r_*′ is negative, we face the so-called Chicken game, which has an internal (polymorphic) equilibrium. If *D_g_*′ is negative and *D_r_*′ is positive, the game, characterized by bi-stability, is called the SH game. Finally, if both *D_g_*′ and *D_r_*′ are negative, we deal with the Trivial game, whereby (C) dominates (D) (i.e. no dilemma exists). Therefore, we can evaluate the evolution of cooperation more precisely if we quantitatively compare the two constitutional strengths of the reciprocity mechanisms in all 2 × 2 games (irrespective of the reciprocity mechanisms and finiteness properties) using a RAD–GID diagram that consists of the two standardized measures (figure 1*a*; electronic supplementary material, table S2). According to the concept of universal scaling, the relaxation of these two types of dilemma is expressed by the shift of the *x*-axis (i.e. the RAD-axis) and the *y*-axis (i.e. the GID-axis) of the RAD–GID phase-plane diagram to the positive domain [20]. In this paper, we refer to the *D_r_*′–*D_g_′* phase diagram without reciprocity as the ‘default' (figure 2*a*). Note that in the RAD–GID phase diagram, the first, second, third and fourth quadrants represent the PD, Chicken, Trivial and SH game structures, respectively (figure 1*a*; electronic supplementary material, table S1).

Here, we apply the five reciprocities (direct reciprocity, indirect reciprocity, kin selection, group selection and network reciprocity) to a traditional (well-mixed infinite population) 2 × 2 game and analyse how these five reciprocities relax the dilemma strength using universal scaling (i.e. the RAD–GID phase planes). We also examine what types of games relax which types of dilemma strength following the introduction of the five reciprocities.

## 2. Model

We verify the dilemma strength of a 2 × 2 game involving a payoff matrix (equation (1.1)). We assume an infinite, well-mixed population (i.e. an infinite number of agents). Two individuals (players) are selected from an unlimited population at random and asked to play the game. Players receive a reward depending on the selected strategies (C) or (D) (equation (1.1)). By introducing the concept of five rules into pair-wise games, we compare the revised (recalculated) dilemma strength of a game that applies one of the five reciprocity rules (*D_r_′*_rev_ and *D_g_′*_rev_) with that of the default game (i.e. *D_r_*′ and *D_g_*′) (see hereafter equations (3.1)–(3.16)).

## 3. Methods

We calculate all the coordinates that are transcribed by each of five reciprocity rules (figure 2). For example, the payoff matrix of direct reciprocity is derived as follows [12,20].

### 3.1. Direct reciprocity

3.1 $$\left(\begin{array}{cc} \frac{R}{1-w} & S+\frac{wP}{1-w}\\ T+\frac{wP}{1-w} & \frac{P}{1-w} \end{array}\right).$$

Note that *w* is the probability of two players meeting each other in another round. The coordinates (*D_r_*′, *D_g_*′) are transferred to (*D_r_*′_rev_, *D_g_*′_rev_) by direct reciprocity as follows:

3.2 $$D_{g\mathrm{rev}}^{\prime }=\frac{\left(T+wP/(1-w))-(R/(1-w)\right)}{\left(R/(1-w))-(P/(1-w)\right)}=f(T,\;R,\;P)=f(D_{g}^{\prime },\;R,\;P)$$

and

3.3 $$D_{r\mathrm{rev}}^{\prime }=\frac{\left(P/(1-w))-(S+wP/(1-w)\right)}{\left(R/(1-w))-(P/(1-w)\right)}=g(S,\;R,\;P)=g(D_{r}^{\prime },\;R,\;P).$$

When *R* = 3 and *P* = 2, *T* and *S* are derived from equations (1.9) and (1.10) by the values of *D_r_*′ and *D_g_*′. Therefore, the coordinate (*D_r_*′, *D_g_*′) = (1, 1) moves to the coordinate (*D_r_*′_rev_, *D_g_*′_rev_) = (0.8, 0.6) by direct reciprocity (see figures 1*b* and 2*b*). Here, we also show the derivations of the dilemma strength of a game that applies one of the other four reciprocity rules (i.e. indirect reciprocity, kin selection, group selection and network reciprocity) as follows.

### 3.2. Indirect reciprocity

3.4 $$\left(\begin{array}{cc} R & (1-q)S+qP\\ (1-q)T+qP & P \end{array}\right).$$

Note that *q* is the probability of knowing the reputation of another individual. The coordinates (*D_r_*′, *D_g_*′) are transferred to (*D_r_*′_rev_, *D_g_*′_rev_) by indirect reciprocity as follows:

3.5 $$D_{g\;\mathrm{rev}}^{\prime }=\frac{\{(1-q)T+qP\}-R}{R-P}=f(T,\;R,\;P)=f(D_{g}^{\prime },\;R,\;P)$$

and

3.6 $$D_{r\;\mathrm{rev}}^{\prime }=\frac{\left\{(1-q)T+qP\}-\{(1-q)S+qP\right\}}{R-P}=g(S,\;R,\;P)=g(D_{r}^{\prime },\;R,\;P).$$

### 3.3. Kin selection

3.7 $$\left(\begin{array}{cc} R & \frac{S+rT}{1+r}\\ \frac{T+rS}{1+r} & P \end{array}\right).$$

Note that *r* is the average relatedness between interacting individuals. The coordinates (*D_r_*', *D_g_*’) are transferred to (*D_r_*′_rev_, *D_g_*′_rev_) by kin selection as follows:

3.8 $$D_{g\;\mathrm{rev}}^{\prime }=\frac{(T+rS)/(1+r)-R}{R-P}=f(T,\;R,\;P)=f(D_{g}^{\prime },\;R,\;P)$$

and

3.9 $$D_{r\;\mathrm{rev}}^{\prime }=\frac{P-(S+rT)/(1+r)}{R-P}=g(S,\;R,\;P)=g(D_{r}^{\prime },\;R,\;P).$$

### 3.4. Group selection

3.10 $$\left(\begin{array}{cc} (m+n)R & nS+mR\\ nT+mP & (m+n)P \end{array}\right).$$

Note that *m* is the number of groups and *n* is the maximum size of a group. The coordinates (*D_r_*', *D_g_*’) are transferred to (*D_r_*′_rev_, *D_g_*′_rev_) by group selection as follows:

3.11 $$D_{g\;\mathrm{rev}}^{\prime }=\frac{(nT+mP)-(m+n)\;R}{(m+n)\;R-(m+n)\;P}=f(T,\;R,\;P)=f(D_{g}^{\prime },\;R,\;P)$$

and

3.12 $$D_{r\;\mathrm{rev}}^{\prime }=\frac{\left(m+n)P-(nS+mR\right)}{(m+n)R-(m+n)P}=g(S,\;R,\;P)=g(D_{r}^{\prime },\;R,\;P).$$

### 3.5. Network reciprocity

3.13 $$\left(\begin{array}{cc} R & S+H\\ T-H & P \end{array}\right).$$

The term *H* is defined as follows:

3.14 $$H=\frac{(k+1)(R-P)-T+S}{\left(k+1)(k-2\right)}.$$

Note that *k* is the number of neighbours. The coordinates (*D_r_*′, *D_g_*′) are transferred to (*D_r_*′_rev_, *D_g_*′_rev_) by direct reciprocity as follows:

3.15 $$D_{g\;\mathrm{rev}}^{\prime }=\frac{(T-H)-R}{R-P}=f(T,\;R,\;P)=f(D_{g}^{\prime },\;R,\;P)$$

and

3.16 $$D_{r\;\mathrm{rev}}^{\prime }=\frac{P-(S+H)}{R-P}=g(S,\;R,\;P)=g(D_{r}^{\prime },\;R,\;P).$$

## 4. Analytical results

Because the introduction of a reciprocity protocol changes the dilemma strengths, it transfers the coordinates (*D_r_*′, *D_g_*′) of the default game to the new coordinates (*D_r_*′_rev_, *D_g_*′_rev_) (figure 1*b–e*; electronic supplementary material, table S3). For example, a default PD (i.e. (*D_r_*′, *D_g_*′) = (0.1, 0.1)) is transferred into the regions of SH or Trivial via dilemma relaxations (figure 1*b*). The five reciprocity rules proceed with the following three types of game-class changes and their combinations:

1. The PD game becomes Chicken via the reduction of RAD (*D_r_*′ decreases to *D_r_*′_rev_).
2. The PD game becomes SH via the reduction of GID (*D_g_*′ decreases to *D_g_*′_rev_).
3. The PD game becomes Trivial (i.e. the dilemma is eliminated) via the reduction of both RAD and GID (figure 1; electronic supplementary material, tables S3 and S4). Note that we can observe both the relaxation of dilemmas and their enhancements (e.g. indirect reciprocity in figure 1*c*–*e*).

Below, we describe the transformation of the default phase plane via the introduction of the five reciprocity rules (figure 2). We can observe the relaxation of positive values of GID and RAD in all five rules (figure 2*b*–*f*). The enhancement of negative values of GID and RAD is also observed (figure 2*b*–*d*). For example, kin selection results in both relaxation and enhancement of negative values of GID and RAD by distorting the square frame (figure 2*d*). Distortion of the frame occurs in kin selection and network reciprocity (figure 1*d*,*f*) because both parameters *S* and *T* appear in single cells of the payoff matrix (see equations (3.7) and (3.13)).

**Figure 3.**
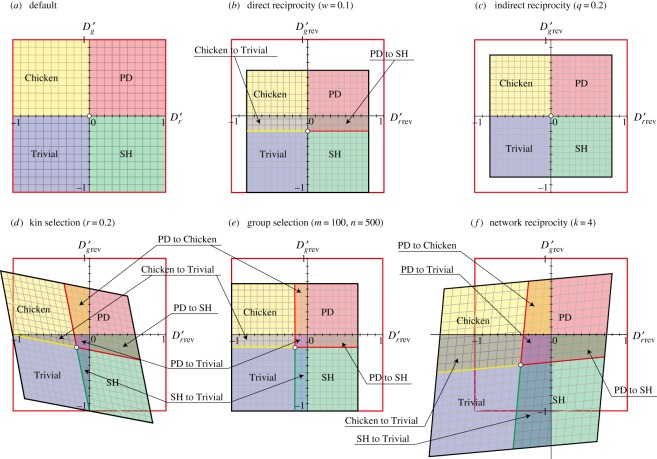
Game-class changes with the introduction of the five reciprocity rules. (*a*) Default phase plane. (*b*–*f*) *D_r_*′_rev_–*D_g_*′_rev_ phase diagram after introducing (*b*) direct reciprocity, (*c*) indirect reciprocity, (*d*) kin selection, (*e*) group selection and (*f*) network reciprocity (see figures 1 and 2 for terminology).

The relaxation of the two dilemmas may or may not result in changes in game classes (figure 3; electronic supplementary material, table S4). In direct reciprocity, because the default origin moves in the opposite direction of the GID, a portion of the Chicken and PD area is converted into Trivial and SH, respectively (figure 3*b*). By contrast, no game-class change is found in indirect reciprocity (figure 3*c*). In the remaining three rules (kin selection, group selection and network reciprocity), because the default origin moves in the opposite direction of GID and RAD, five types of game-class conversion occur (i.e. PD to Chicken, PD to Trivial, PD to SH, Chicken to Trivial and SH to Trivial) (figure 3*d*–*f*; electronic supplementary material, table S4). The regions of the game-class change are increased as the strength of social viscosity increases (electronic supplementary material, figures S1–S5).

## 5. Discussion

Previous studies of universal scaling parameters quantitatively estimate the strengths of dilemma relaxation [10–13]. In this study, by introducing the graphical representation of dilemma strengths, we analyse the qualitative differences in the dilemma relaxation in a pair-wise game when five reciprocity rules are introduced. We visually demonstrate that the five rules have different mechanisms for eliminating dilemmas by distorting/transforming the dilemma phase plane. We also show where the conversion of game classes occurs in the phase-plane diagram in each of the five cases.

The current graphical approach may be useful for interpretations of social cooperation in game-theory contexts. Using this approach, we can divide these five rules into three categories of reciprocity promoters: (i) originator (direct reciprocity), (ii) potentiator (indirect reciprocity) and (iii) booster/enhancer (kin selection, group selection, network reciprocity). First, direct reciprocity induces the phase changes along GID (Chicken to Trivial and PD to SH), but no phase change occurs along RAD. Direct reciprocity is often ensured by group living (e.g. nest-dwelling), which is necessary for the origin of cooperation [2,3,21–23]. The category of indirect reciprocity does not produce phase conversion (game-class change), even though it potentiates the other rules by shrinking the dilemma phase space [21,24,25]. Note that in indirect reciprocity, the dilemma strength can be increased for the Chicken or SH games (figure 2*c*). The third category is the booster/enhancer of three different types. Kin selection is the enhancer of cooperation in the colony of close kin, leading to eusociality [25–30]. The close kin group is easily formed in underground group-living dwellers, e.g. termites, naked moles, wasps/bees and ants [31,32], and haplodiploidy should have further promoted the eusociality in hymenopterans [33]. Group selection has a strong effect in enhancing cooperative behaviour in any society, including non-kin groups. Group selection is suspected to develop in extremely harsh environments (e.g. extremely cold climates, extremely high elevation) because the persistence of an entire group is threatened [34]. For example, lichen has evolved obligate symbiosis in the air or on rock, where no other organisms can sustain themselves. Stochastic environments may also promote cooperation for the same reason [35].

Both group selection and network reciprocity are suspected as a promotion function in human societies [34,36,37]. These two reciprocity mechanisms have an effect as a booster/enhancer of cooperative behaviour in societies in which cooperation has fully penetrated. All of the reciprocity mechanisms in the third category induce phase changes (game-class changes) along both GID and RAD (figures 1–3). Therefore, we can expect that these three types of reciprocity mechanisms are a strong booster/enhancer for the promotion of cooperation in developing societies.

## Supplementary Material

Supplementary material of "Scaling the phase- planes of social dilemma strengths shows game-class changes in the five rules governing the evolution of cooperation"

## Data accessibility

The authors declare that all data supporting the findings of this study are available within the article and its electronic supplementary material files or from the corresponding author upon reasonable request.
