# Correction to ‘Scaling the phase-planes of social dilemma strengths shows game-class changes in the five rules governing the evolution of cooperation’

**DOI:** 10.1098/rsos.200943

**Published:** 2020-06-17

**Authors:** Hiromu Ito, Jun Tanimoto

*R. Soc. Open Sci.* **5**, 181085. (Published online 17 October 2018). (doi:10.1098/rsos.181085)

Please see the below summary of corrections. These have now been corrected.

The transcription of GID-axis in indirect reciprocity was wrongly displayed. As a result of our recalculation, indirect reciprocity has exactly the same payoff matrix with direct reciprocity, indicating the same dilemma relaxation mechanism. Then, indirect reciprocity has a potential to change game class as in direct reciprocity. Therefore, all five rules (i.e. direct reciprocity, indirect reciprocity, kin selection, group selection and network reciprocity) have the potential to change game class. Direct reciprocity and indirect reciprocity only relax GID (gamble-intending dilemma), the other three rules relax GID and RAD (risk-averting dilemma). Thus, Nowak's five reciprocity rules include four (not five) dilemma relaxation mechanisms.

The five reciprocity rules should be divided into two categories of reciprocal promoters: (i) originator (direct reciprocity and indirect reciprocity) and (ii) booster/enhancer (kin selection, group selection, network reciprocity). Since GID means the strength of the dilemma trying to exploit each other, relaxation of GID is more effective in promoting cooperative behaviour than RAD. Thus, indirect reciprocity also functions as a strong reciprocity mechanism as direct reciprocity.

**Correction 1**

**Abstract**

In this work, by drawing a RAD–GID phase-plane diagram for pair-wise games, we prove that these five rules are indeed quite different for the resolution (relaxation) of the two dilemmas.

**Correct text:**

In this work, by drawing a RAD–GID phase-plane diagram for pair-wise games, we prove that these five rules have four different dilemma resolution (relaxation) mechanisms.

**Correction 2**

**Analytical results**

3. The PD game becomes Trivial (i.e. the dilemma is eliminated) via the reduction of both RAD and GID (figure 1; electronic supplementary material, tables S3 and S4). Note that we can observe both the relaxation of dilemmas and their enhancements (e.g. indirect reciprocity in figure 1*c*–*e*).

**Correct text:**

3. The PD game becomes Trivial (i.e. the dilemma is eliminated) via the reduction of both RAD and GID (figure 1; electronic supplementary material, tables S3 and S4). Note that we can observe not only relaxation of GID but also enhancements of RAD in direct reciprocity and indirect reciprocity (figure 1*d*,*e*).

**Correction 3**

The relaxation of the two dilemmas may or may not result in changes in game classes (figure 3; electronic supplementary material, table S4). In direct reciprocity, because the default origin moves in the opposite direction of the GID, a portion of the Chicken and PD area is converted into Trivial and SH, respectively (figure 3b). By contrast, no game-class change is found in indirect reciprocity (figure 3*c*). In the remaining three rules.

**Correct text:**

The relaxation of the two dilemmas may or may not result in changes in game classes (figure 3; electronic supplementary material, table S4). In direct reciprocity and indirect reciprocity, because the default origin moves in the opposite direction of the GID, a portion of the Chicken and PD area is converted into Trivial and SH, respectively (figure 3*b*,*c*). In the remaining three rules.

**Correction 4**

**Discussion**

We visually demonstrate that the five rules have different mechanisms for eliminating dilemmas by distorting/transforming the dilemma phase plane.

**Correct text:**

We visually demonstrate that the five rules have four different mechanisms for eliminating dilemmas by distorting/transforming the dilemma phase plane.

**Correction 5**

Using this approach, we can divide these five rules into three categories of reciprocity promoters: (i) originator (direct reciprocity), (ii) potentiator (indirect reciprocity) and (iii) booster/enhancer (kin selection, group selection, network reciprocity). First, direct reciprocity induces the phase changes along GID (Chicken to Trivial and PD to SH), but no phase change occurs along RAD. Direct reciprocity is often ensured by group living (e.g. nest-dwelling), which is necessary for the origin of cooperation [2,3,21–23]. The category of indirect reciprocity does not produce phase conversion (game-class change), even though it potentiates the other rules by shrinking the dilemma phase space [21,24,25]. Note that in indirect reciprocity, the dilemma strength can be increased for the Chicken or SH games (figure 2c). The third category is the booster/enhancer of three different types. Kin selection is the enhancer of cooperation in the colony of close kin, leading to eusociality [25–30].

**Correct text:**

Using this approach, we can divide these five rules into two categories of reciprocity promoters: (i) originator (direct reciprocity and indirect reciprocity) and (ii) booster/enhancer (kin selection, group selection, network reciprocity). First, direct reciprocity and indirect reciprocity induce the phase changes along GID (Chicken to Trivial and PD to SH), but no phase change occurs along RAD. Direct reciprocity and indirect reciprocity are often ensured by group living (e.g. nest-dwelling), which is necessary for the origin of cooperation [2,3,21–25]. The second category is the booster/enhancer of three different types. Kin selection is the enhancer of cooperation in the colony of close kin, leading to eusociality [25–30].

**Correction 6**

All of the reciprocity mechanisms in the third category induce phase changes (game-class changes) along both GID and RAD (figures 1–3). Therefore, we can expect that these three types of reciprocity mechanisms are a strong booster/enhancer for the promotion of cooperation in developing societies.

**Correct text:**

All of the reciprocity mechanisms in the second category (i.e., kin selection, group selection, network reciprocity) induce phase changes (game-class changes) along both GID and RAD (figures 1–3). Therefore, we can expect that these three types of reciprocity mechanisms are a strong booster/enhancer for the promotion of cooperation in developing societies.

**Correction 7**

**An additional reference has been added:**

38. Tanimoto J. 2018 *Evolutionary games with sociophysics*. Singapore: Springer.

**Correction 8**

In figures 1, 2 and 3, the parameter of direct reciprocity (*w*: the probability of two players meeting each other in another round) is wrong. The correct parameter is *w* = 0.2.

**Correction 9**

In figure 1, 2 and 3, the transcribe of GID-axis in indirect reciprocity is wrong. Indirect reciprocity has the completely same coordinate transformation effect as direct reciprocity (see also [38]). In direct reciprocity and indirect reciprocity, because the default origin moves in the opposite direction of the GID, a portion of the Chicken and PD area is converted into Trivial and SH, respectively.

The correct figures show as follows:

**Figure 1.**
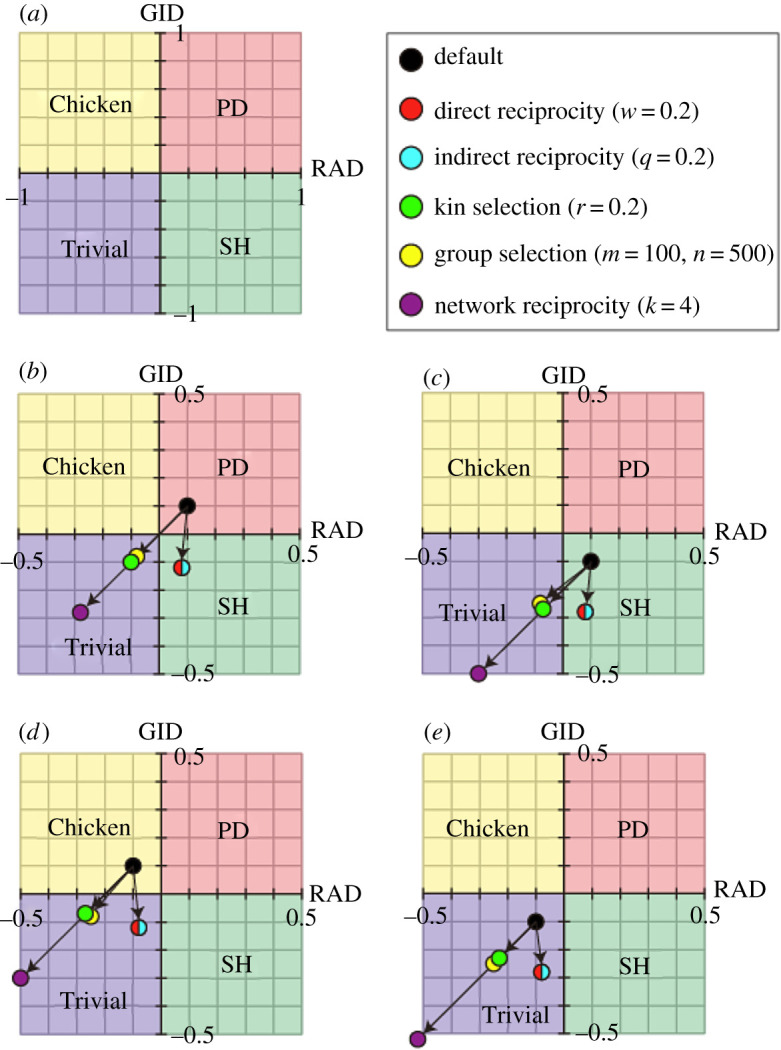
Phase planes of a pair-wise game with coordinate movements with the introduction of the five reciprocity rules. The shaded colours indicate the regions of Trivial (blue), Prisoner's dilemma (PD) (red), Chicken (yellow) and Stag-hunt (SH) (green) games. (*a*) Default RAD–GID phase plane. (*b*–*e*) Phase plane with coordinate movements according to the five rules. The default point (black circle) moves to each point (see above keys) with the introduction of the five rules. Initial default coordinates (*D_r_*’, *D_g_*’) are (*b*) (0.1, 0.1) in PD, (*c*) (0.1, −0.1) in SH, (*d*) (−0.1, 0.1) in Chicken, and (*e*) (−0.1, −0.1) in Trivial.

**Figure 2.**
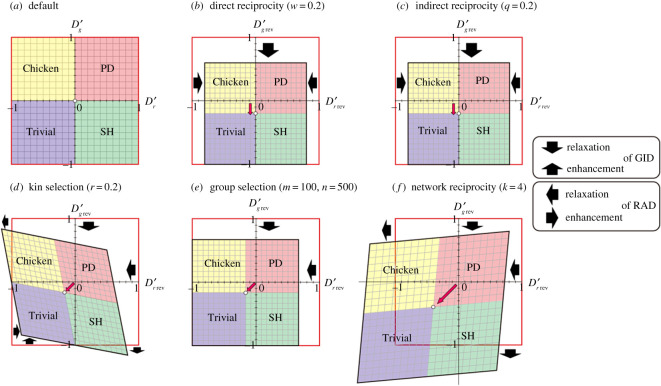
Transformation of the default phase plane (–1 ≤ *D_g_*’, *D_r_*’ ≤ +1) with the introduction of the five rules in all 2 × 2 games. The shaded colours indicate the regions of Trivial (blue), Prisoner's dilemma (PD) (red), Chicken (yellow) and Stag-hunt (SH) (green) games. (*a*) Default phase plane. (*b*–*f*) Transformed phase plane with the introduction of (*b*) direct reciprocity, (*c*) indirect reciprocity, (*d*) kin selection, (*e*) group selection and (*f*) network reciprocity. The origin in the default moves to the direction of the points indicated by pink arrows. The red flames indicate the default phase planes. The thick black arrows indicate the relaxation and enhancement of GID and RAD.

**Figure 3.**
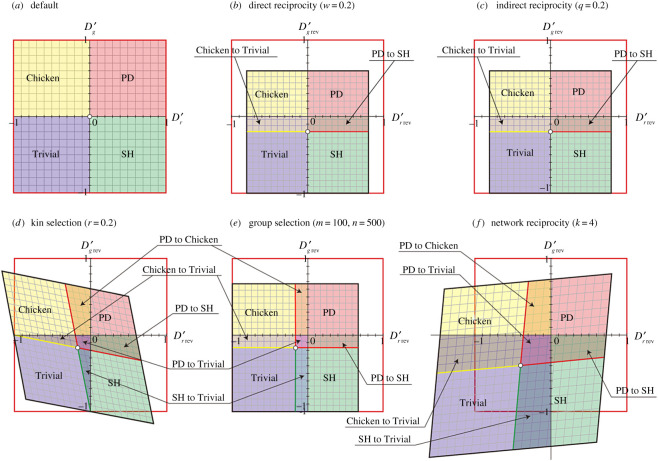
Game-class changes with the introduction of the five reciprocity rules. (*a*) Default phase plane. (*b*–*f*) *D_r_*’*_rev_*– *D_g_*’ *_rev_* phase diagram after introducing (*b*) direct reciprocity, (*c*) indirect reciprocity, (*d*) kin selection, (*e*) group selection and (*f*) network reciprocity (see figures 1 and 2 for terminology).

