# Supplementary material for: Scaling the phase-planes of social dilemma strengths shows game-class changes in the five rules governing the evolution of cooperation
Source: R Soc Open Sci. 2018 Oct 17;5(10):181085. doi: 10.1098/rsos.181085 (PMC6227953; doi:10.1098/rsos.181085)
Supplement: Supplementary material of "Scaling the phase- planes of social dilemma strengths shows game-class changes in the five rules governing the evolution of cooperation" [file rsos181085supp1.pdf]

Supplementary Information

Scaling the phase- planes of social dilemma strengths shows game-class changes in the five rules governing the evolution of cooperation

Hiromu Ito and Jun Tanimoto

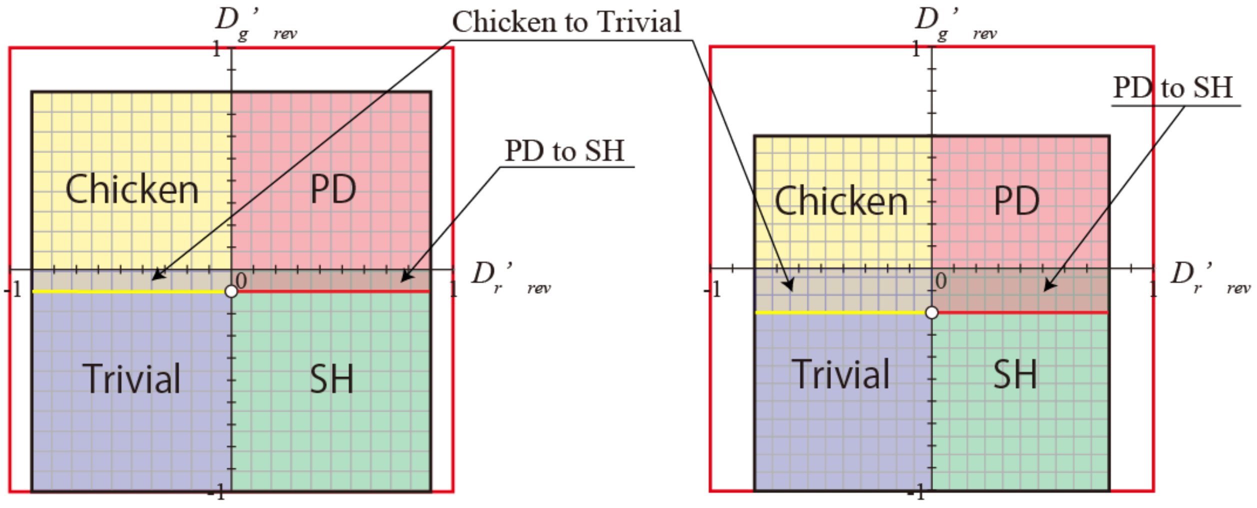

Supplementary Figure S1. Distribution of dilemma strength and the strength of direct reciprocity. The probability of meeting each other in another round is x = 0.2 (left) and x = 0.1 (right).

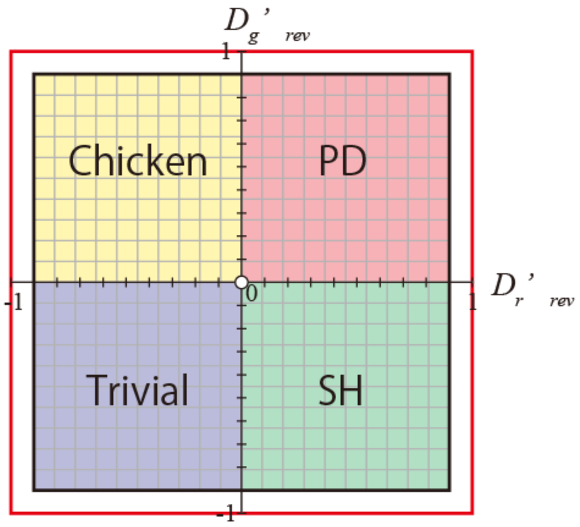

Supplementary Figure S2. Distribution of dilemma strength and the strength of indirect reciprocity. The probability of knowing the reputation of another individual is q = 0.1 (left) and q = 0.2 (right).

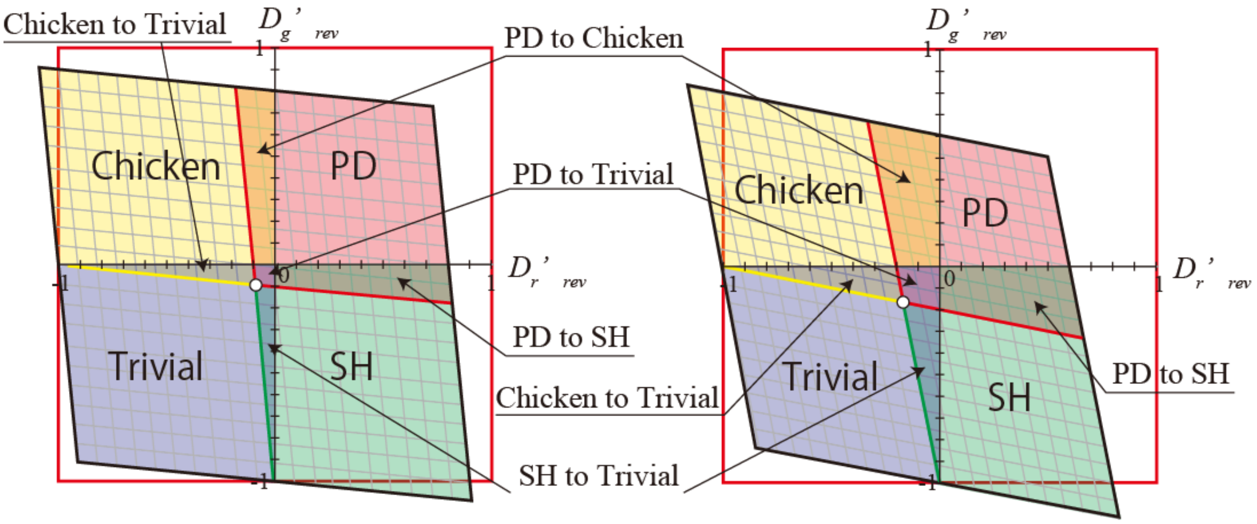

Supplementary Figure S3. Distribution of dilemma strength and the strength of kin selection. The average relatedness of interacting individuals is r = 0.1 (left) and r = 0.2 (right).

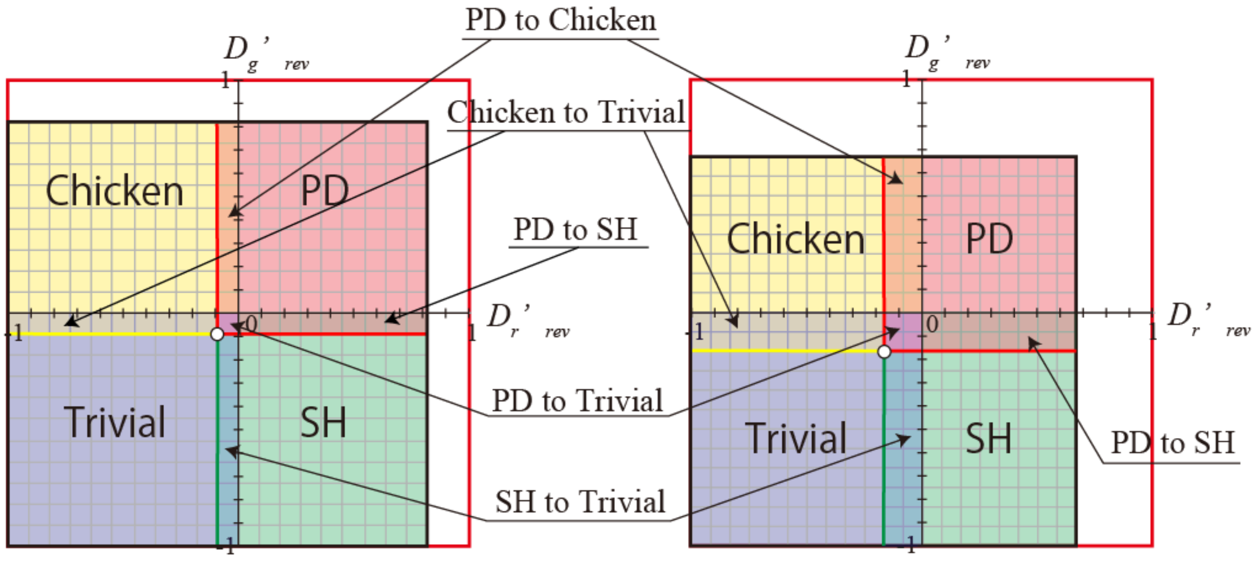

Supplementary Figure S4. Distribution of dilemma strength and the strength of group selection. The number of groups is m = 50 (left) and m = 100 (right), and the maximum size of a group is n = 500.

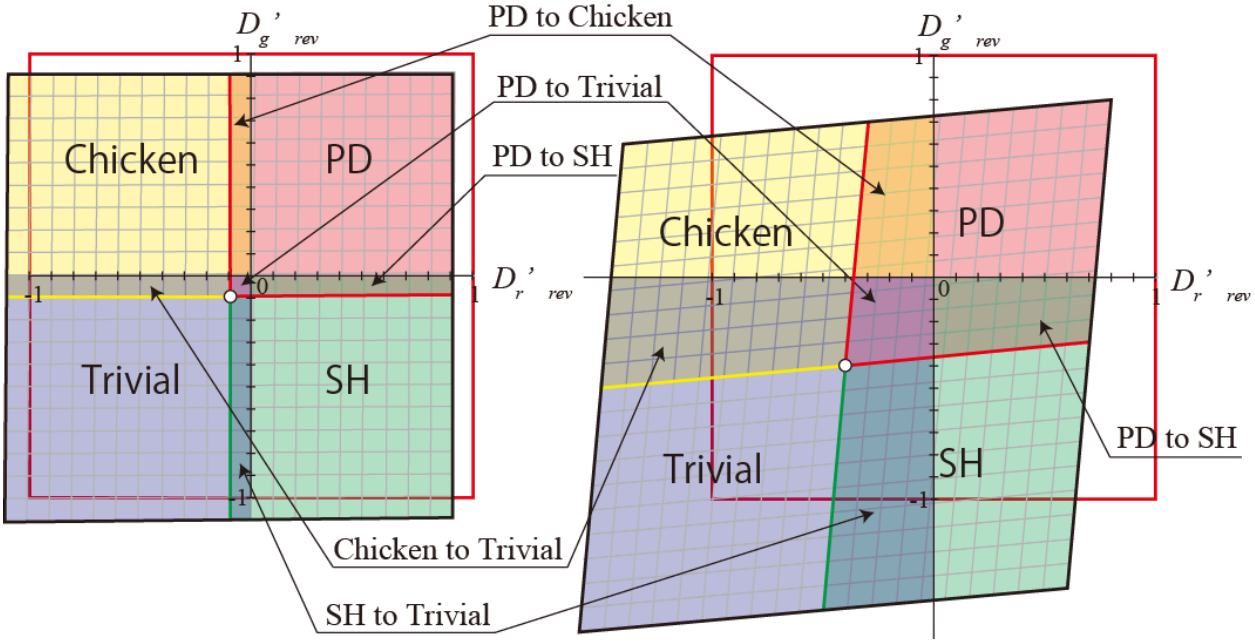

Supplementary Figure S5. Distribution of dilemma strength and the strength of network reciprocity. The number of neighbours is k = 12 (left) and k = 4 (right).

Supplementary Table S1. Class types in 2 × 2 games.

Game class	Existence of dilemma	GID	RAD
Prisoner’s dilemma; PD	Positive	Positive	Positive
Chicken (Snowdrift; Hawk-dove)	Positive	Positive	Negative
Stag hunt; SH	Positive	Negative	Positive
Trivial	Negative	Negative	Negative

Supplementary Table S2. List of parameters.

	Parameter	Description
	x	Probability of meeting each other in another round (direct reciprocity)
	q	Probability of knowing the reputation of another individual (indirect reciprocity)
	r	Average relatedness of interacting individuals (kin selection)
	m	Number of groups (group selection)
	n	Maximum size of group (group selection)
	k	Number of neighbours (network reciprocity)
GID: Gamble-intending dilemma	D_g	Dilemma strength of GID (classic)
	D_g'	Refined dilemma strength of RAD in default game
	$D_g{}'_{rev}$	Refined dilemma strength of GID in game with reciprocity mechanisms
RAD: Risk-averting dilemma	D_r	Dilemma strength of RAD (classic)
	D_r'	Refined dilemma strength of RAD in default game
	$D_r{}'_{rev}$	Refined dilemma strength of RAD in game with reciprocity mechanisms

Supplementary Table S3. Detail of dilemma relaxation and game class conversion from four coordinates in Figure 1

	Fig. 1b	
	(D_r', D_g')	Game class
Default	(0.1, 0.1)	PD
	$({D_r}'_{rev}, {D_g}'_{rev})$	
Direct reciprocity	(0.08, -1.2)	SH
Indirect reciprocity	(0.08, 0.08)	PD
Kin selection	(-0.1, -0.1)	Trivial
Group selection	(-0.08, -0.08)	Trivial
Network reciprocity	(-0.28, -0.28)	Trivial
	Fig. 1c	
	(D_r', D_g')	Game class
Default	(0.1, -0.1)	SH
	$({D_r}'_{rev}, {D_g}'_{rev})$	
Direct reciprocity	(0.08, -0.28)	SH
Indirect reciprocity	(0.08, -0.08)	SH
Kin selection	(-0.07, -0.27)	Trivial
Group selection	(-0.08, -0.25)	Trivial
Network reciprocity	(-0.3, -0.5)	Trivial
	Fig. 1d	
	(D_r', D_g')	Game class
Default	(-0.1, 0.1)	Chicken
	$({D_r}'_{rev}, {D_g}'_{rev})$	
Direct reciprocity	(-0.08, -0.12)	Trivial
Indirect reciprocity	(-0.08, 0.08)	Chicken
Kin selection	(-0.27, -0.07)	Trivial
Group selection	(-0.25, -0.08)	Trivial
Network reciprocity	(-0.28, -0.28)	Trivial
	Fig. 1e	
	(D_r', D_g')	Game class

Default	(-0.1, -0.1)	Trivial
	($D_r'_{rev}$, $D_g'_{rev}$)	
Direct reciprocity	(-0.08, -0.28)	Trivial
Indirect reciprocity	(-0.08, -0.08)	Trivial
Kin selection	(-0.23, -0.23)	Trivial
Group selection	(-0.25, -0.25)	Trivial
Network reciprocity	(-0.52, -0.52)	Trivial

Supplementary Table S4. Dilemma relaxation and game class conversion.

		Movement of game class boundary		Game class conversion
		RAD (D_r')	GID (D_g')	
(a)	Direct reciprocity	N/A	Positive	Chicken→Trivial, PD→SH
(b)	Indirect reciprocity	N/A	N/A	N/A
(c)	Kin selection	Positive	Positive	Chicken→Trivial, PD→SH PD→Chicken, PD→Trivial, SH→Trivial
(d)	Group selection	Positive	Positive	Chicken→Trivial, PD→SH PD→Chicken, PD→Trivial, SH→Trivial
(e)	Network reciprocity	Positive	Positive	Chicken→Trivial, PD→SH PD→Chicken, PD→Trivial, SH→Trivial
